# Salivary Human β-Defensin 1-3 and Human α-Defensin-1 Levels in Relation to the Extent of Periodontal Disease and Tooth Loss in the Elderly

**DOI:** 10.3390/jcm12030976

**Published:** 2023-01-27

**Authors:** Ulvi Kahraman Gürsoy, Mervi Gürsoy, Anna Liukkonen, Anna Liisa Suominen, Eija Könönen

**Affiliations:** 1 Department of Periodontology, Institute of Dentistry, University of Turku, 20500 Turku, Finland; 2 Welfare Division, Oral Health Care, 20101 Turku, Finland; 3 Finnish Institute for Health and Welfare, 00271 Helsinki, Finland; 4 Institute of Dentistry, University of Eastern Finland, 70211 Kuopio, Finland; 5 Oral Health Services, 33101 City of Tampere, Finland

**Keywords:** aged, alpha-defensins, beta-defensins, saliva

## Abstract

The oral innate immune response may diminish with aging. In the present study, the aim was to examine human β-defensin (hBD) 1-3 and human neutrophil peptide (HNP)-1 levels in the saliva of an elderly population to establish the extent of periodontal disease and tooth loss. A total of 175 individuals aged ≥ 65 years were divided into five groups based on the number of teeth with a pocket depth ≥ 4 mm as follows: 17 pocket-free individuals (Control), 55 individuals having 1–6 pocket teeth (PerioA), 33 individuals having 7–13 pocket teeth (PerioB), 29 individuals having at least 14 pocket teeth (PerioC), and 41 edentulous individuals. Their salivary defensin levels were measured with ELISA kits. The salivary HNP-1 levels were significantly higher in the Perio groups (PerioB: *p* < 0.001 and PerioC: *p* < 0.001) in comparison to the Control. The associations between salivary HNP-1 levels and the number of pocket teeth remained significant after adjustments for age, gender, level of education, and number of teeth. The salivary HNP and hBD levels differed in terms of their correlation to the extent of periodontal disease and tooth loss in the elderly.

## 1. Introduction

Oral cavities harbor both commensal and pathogenic bacteria at all times, having a constant interaction with host cells. The homeostasis between the oral microbiome and the host is maintained mainly by the components of the oral innate and adaptive immune responses [1]. Defensins, which are small cationic peptides with broad antimicrobial and chemotactic activities, play an essential role in controlling the oral environment. Epithelial (human β-defensins, hBD) and neutrophilic (α-defensins, human neutrophilic peptide, HNP) defensins are located in oral mucosae [2,3] and can be detected in saliva and gingival crevicular fluid [4,5]. hBD-1 is constantly expressed in the oral cavity, while the expressions of hBD-2 and hBD-3 are dependent on the extent of infection and inflammation. Indeed, the localizations of oral mucosal defensins during health also differ; while HNP-1 is localized in the junctional epithelium, hBD-2 is localized in the superficial layers and hBD-3 is localized in the basal layers of the oral and sulcular epithelium [2,6]. Finally, whereas the oral commensal bacteria stimulate the expressions of these antimicrobial peptides, the periodontitis-associated bacteria are able to suppress their expression or to degrade defensins with bacterial proteases [6,7].

Various changes and disorders in the immune response, namely immunosenescence, occur in the human body with aging [8,9]. For example, it has been shown that the receptor expression and signal transduction pathways are disturbed in phagocytic cells [10] and HNP-1 production of neutrophils is decreased [11] with age. In the elderly, an age-related decrease in neutrophil extracellular trap formation has been observed in periodontitis patients [12]. Moreover, aged people with gingivitis show increased expressions of interleukin (IL)-1β and IL-6 [13]. There are few studies exploring the oral hBD-or HNP-expression profiles in the elderly. Reduced salivary hBD-2 levels found in the elderly compared to young individuals [14] differ from the conserved hBD-2 levels found in serum with aging [15]. However, an interesting observation of the age-related difference in the localization of hBD-2 in human gingiva [16] indicates that the oral and systemic hBD expressions are regulated differently. Nevertheless, the intraoral expression profiles of hBD 1-3 and HNP-1 in relation to the extent of periodontal destruction in the elderly are still unknown.

In aged people, tooth loss is a common event that reflects compromised health conditions of teeth and/or tooth-supporting tissues. Indeed, the extent of tooth loss is considered a surrogate measure of past, but also present, oral disease(s). Periodontal disease is one typical reason for missing teeth in adult-aged populations [1]. It is notable that the destruction of tooth-supporting tissues proceeds without noticeable signs until considerably loosened attachment, meaning that periodontitis-affected teeth may stay for years without treatment. Consequently, the infectious and inflammatory burden in the mouth can be high. 

Recently, our group demonstrated that salivary hBD-2 and hBD-3 levels do not relate to the extent of gingival inflammation in children and adolescents [17] nor in adults with periodontitis [18]. To our knowledge, however, there is no study in the literature to compare the expression profile of oral hBDs and HNPs in aged people in this context. In this study, we hypothesized that the response of immune cells is reduced with age, and thus, intraoral epithelial hBD and neutrophilic HNP responses differ from each other. Therefore, the aim of the present study was to examine whether salivary profiles of hBD 1-3 and HNP-1 differ from each other in the elderly, in terms of their correlation to the extent of periodontal destruction.

## 2. Materials and Methods

### 2.1. The Study Population and Ethical Permission

The elderly population in the present study was part of a national health survey of the working-aged and elderly Finnish population (the Health 2000 Health Examination Survey), conducted by the Finnish Institute for Health and Welfare (the former National Public Health Institute) during the years 2000–2001. Data on each individual were gathered by questionnaires, an interview at home or in an institute, and health examination, including laboratory tests, in the local health care center or comparable premises. In the southern district premises, an oral specimen (saliva) was collected as part of the laboratory procedures [19]. Written informed consents were collected from the participants in the survey. The study protocols were approved by the Ethical Committee for Epidemiology and Public Health of the Hospital District of Helsinki and Uusimaa, Finland (Trial protocol 407/E3/2000).

The present study includes data on all individuals aged ≥65 years, who participated in questionnaires, interviewing, and clinical general health and oral health examinations, and from whom stimulated saliva samples were collected (*n* = 175). The demographic information, including age, gender, the level of education (basic, secondary, and higher), and smoking history (a daily/occasional smoker or a former (had quit ≥ 1 year ago)/never smoker), originated from questionnaires and interviewing. Measurements for the body mass index (BMI) were registered in the health examination. The information on oral health status included the number of teeth and the number of pocket teeth, i.e., having probing pocket depths (PPDs) ≥ 4 mm, measured by a specialist dentist with the assistance of a dental nurse in a standard dental unit. More detailed information on oral and periodontal examination can be found elsewhere [19,20].

### 2.2. Formation of Study Groups

Altogether 175 individuals aged ≥65 years (105 females, 70 males; mean age 74.35; age range 65–92 years) were divided into five groups based on the number of pocket teeth (PPD ≥ 4 mm) as follows: 17 pocket-free individuals (Control), 55 individuals having 1–6 pocket teeth (PerioA), 33 individuals having 7–13 pocket teeth (PerioB), 29 individuals having at least 14 pocket teeth (PerioC), and 41 edentulous individuals.

### 2.3. Determination of Salivary hBD 1-3 and HNP-1

Paraffin-stimulated whole saliva samples collected by expectoration were immediately frozen in carbonic acid ice for 1 to 3 days before and during the transportation. Thereafter, samples were frozen at −70 °C until they were processed.

For detection of salivary hBD-1 and -2 levels, commercial sandwich- enzyme-linked immunosorbent assay (ELISA) kits (PeproTech^®^, Rocky Hill, CT, USA) were used. In-house sandwich-ELISA assays (Rabbit anti-hBD-3, cat# 500-P241; Biotinylated rabbit anti-hBD-3, cat# 500-P241; hBD-3, cat# 300-52; Goat anti-HNP-1, cat#500-P126G; Biotinylated goat anti-hNP-1, cat# 500-P126G, and hNP-1, cat# 300-42; PeproTech^®^) were used in detections of salivary hBD-3 and HNP-1 levels. A detailed description of the ELISA assays is presented elsewhere [21].

### 2.4. Statistical Analyses

All data analyses were carried out with the SPSS statistical program (version 26.0; IBM Corp., Armonk, NY, USA). In normally distributed parameters (age, number of teeth, and BMI), a two-way analysis of variance (ANOVA) test followed by a post-hoc t-test was applied. Non-parametric Kruskal–Wallis (for multiple comparisons) and Dunn–Bonferroni post hoc methods were used when comparing non-normally distributed parameters (biochemical markers of saliva). The significance values were adjusted by the Bonferroni correction for multiple tests. The Chi-Square test was used to compare the percentage of males and smokers between the groups. A linear regression analysis was used to analyze the associations between the salivary HNP-1 concentrations and the increased number of teeth with PPD ≥ 4 mm (the edentulous group was omitted during the regression analysis), in the presence or absence of confounders. Statistical significance was defined as *p* < 0.05.

## 3. Results

The characteristics of the study population, which was divided into five groups, are presented in Table 1. The PerioA (*p* = 0.015), PerioB (*p* = 0.032), and PerioC (*p* = 0.001) groups were significantly younger than the edentulous participants (Table 1). Being edentulous was especially common in the elderly with a basic educational level only. The PerioB and PerioC groups had more teeth compared to both the Control group (*p* = 0.002 and *p* = 0.001, respectively) and the PerioA group (*p* = 0.001 and *p* < 0.001, respectively) (Table 1). 

**Figure 1:**
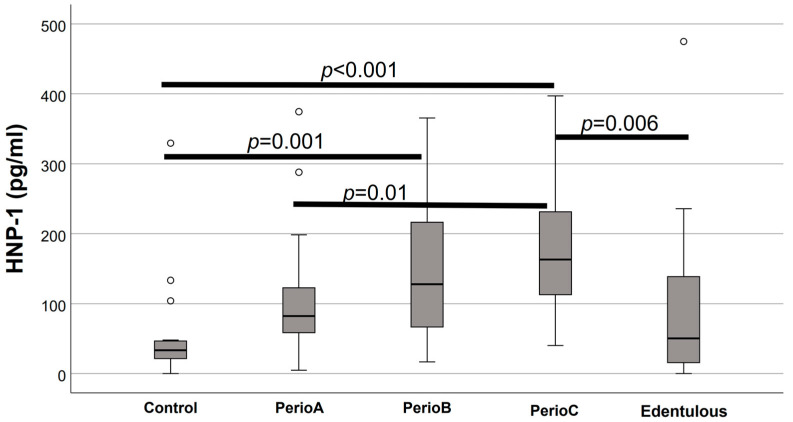
Salivary human neutrophilic peptide (HNP)-1 levels in relation to the extent of periodontal destruction. Significant differences between the groups are marked with connector lines and *p* values are denoted above (the empty circles indicate outliers).

**Figure 2:**
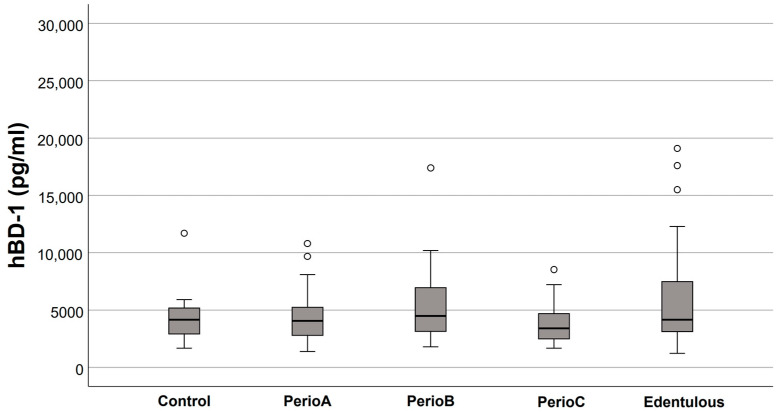
Salivary human β-defensin (hBD)-1 levels in relation to the extent of periodontal destruction (empty circles indicate outliers). No significant differences were found between the groups.

**Figure 3:**
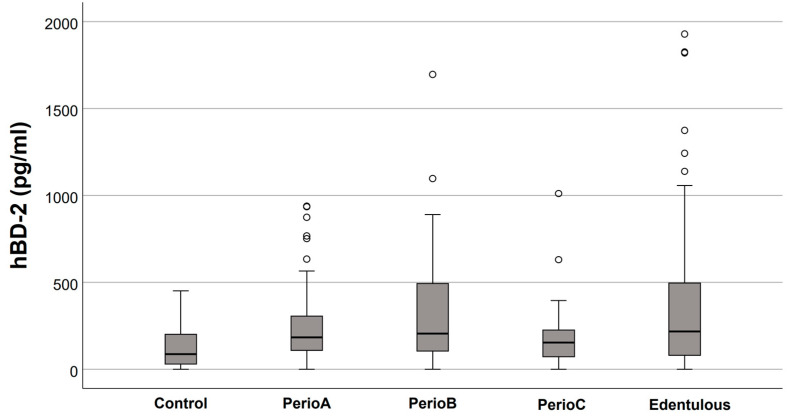
Salivary human β-defensin (hBD)-2 levels in relation to the extent of periodontal destruction (empty circles indicate outliers). No significant differences were found between the groups.

**Figure 4:**
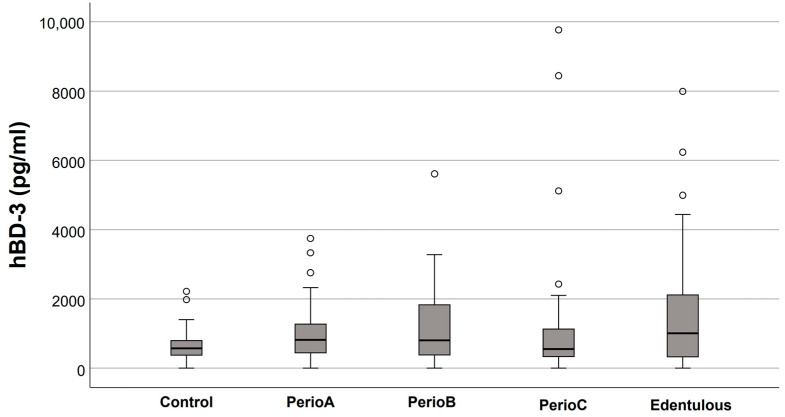
Salivary human-β defensin (hBD)-3 levels in relation to the extent of periodontal destruction (empty circles indicate outliers). No significant differences were found between the groups.

Salivary defensin levels in the five study groups are presented in Figure 1, Figure 2, Figure 3 and Figure 4. Elevated HNP-1 levels were observed in the PerioB (*p* = 0.001) and PerioC (*p* < 0.001) groups compared to the Controls. There were no differences in hBD 1-3 or HNP-1 levels when edentulous participants and Controls were compared.

Linear regression analyses revealed an association between elevated salivary HNP-1 levels and having an increased number of teeth with PPD ≥ 4 mm (Table 2).

## 4. Discussion

To our knowledge, our study is the first to compare the salivary levels of epithelial (hBD) and neutrophilic (HNP) defensins in relation to the periodontal status of an elderly population. Here, we demonstrated that unlike the salivary levels of hBD-1, hBD-2, and hBD-3, those of HNP-1 relate to the extent of periodontal disease in individuals aged 65 years or more. A second novel finding was that the salivary HNP-1 and hBD 1-3 levels of edentulous individuals were close to those of periodontal pocket-free Controls. This finding indicates that in a healthy oral cavity, the salivary defensin levels stay steady even after the extraction of all teeth.

According to the present results, salivary HNP-1 levels elevate gradually with the increased number of pocket teeth. This finding of elevated HNP-1 levels in the periodontally diseased elderly is in line with our recent study on defensin levels in an adult population with an age range of 40–60 years [21]. A further comparison of defensin levels between the latter and present studies revealed that the levels of salivary HNP-1 in the Control group elderly were lower (median 33.4 pg/mL, range 0–329 pg/mL) than those in the adult population (median 81.9 pg/mL, range 17.4–184 pg/mL). On the other hand, a comparison of individuals having at least 14 teeth with PPD ≥ 4 mm, between these two study populations (i.e., corresponding with the PerioC group of the present study and a generalized periodontitis group of the latter study), revealed higher HNP-1 levels in the elderly than in working-aged adults (median 163 pg/mL, range 40.2–397 pg/mL vs. median 103 pg/mL, range 17.4–305 pg/mL, respectively). Thus, the earlier observation that HNP-1 levels are suppressed with aging [11] is supported by our findings only for the individuals without periodontitis. Age does not seem to have an effect on the quantity of circulating neutrophils or on the ability of neutrophils to migrate to the infected site, although antimicrobial mechanisms of neutrophils seem to diminish by age [22]. Despite these observations, the current findings indicate that HNP-1 secretion is activated in connection with periodontal infection in the elderly.

Based on the present study’s results, hBD-2 levels are elevated in saliva where there is an increased extent of periodontitis; however, significant differences disappeared when *p* values were adjusted by the Bonferroni correction for multiple tests. In our recent study on salivary defensins in working-aged adults [21], the highest hBD-2 levels were measured in the localized periodontitis group (PPD ≥ 4 mm at 2–7 teeth), whereas the hBD-2 levels did not differ between the Control and the generalized periodontitis groups (no teeth vs. ≥14 teeth with PPD ≥ 4 mm). It is possible that the decreased hBD-2 levels with the expanded burden of the infection-induced inflammation are related to the enzymatic degradation of hBDs by host-derived and bacterial proteases. In a microbiological study based on the southern Finnish population of the Health 2000 Health Examination Survey [19], it was observed that the salivary detection rates of *Porphyromonas gingivalis*, which is a highly proteolytic periodontal pathogen, increased considerably with age. After adjusting for several variables, the carriage of *P. gingivalis* was found to be approximately 20% in the youngest age group (30–34 years), but increased to over 50% in the two oldest age groups (65–74 years and ≥75 years). Although aging, per se, was significantly associated with increased *P. gingivalis* rates, the factors influencing this age-dependent carriage pattern remained unexplained [19]. Interestingly, ourselves and others have shown that hBDs are prone to proteolytic degradation [23] and an increase in proteolytic activity negatively correlates with hBD-2 levels in vivo [18]. 

In the present study, no significant changes were found between the increased number of pocket teeth and hBD-1 or hBD-3 levels in saliva. The latter defensin is highly related to cellular migration and proliferation, thus, activating wound healing as well [24]; whereas, hBD-1 is constantly expressed in the oral and sulcular epithelium [6,25]. Considering that the density and regeneration ability of the oral epithelium decrease with aging [26], it is possible that hBD-1 and hBD-3 responses in the gingiva are suppressed with age. 

The present study included all ≥65-year-old participants of the southern Finnish population of the Health 2000 Health Examination Survey [19] from whom saliva samples were available; therefore, no sample size determination was performed. Our observation that pocket-free individuals (Controls) had a significantly lower number of teeth than individuals in the PerioB and PerioC groups may be explained by the history of periodontitis treated with extractions in the Control group. As the number of teeth, smoking, and level of education may have direct or indirect effects on salivary hBD levels [6], these parameters were included in the linear regression analysis as covariants. It was observed that individuals with a basic level of education were more often edentulous than the more educated individuals, which is potentially due to differences in self-care attitudes and the cumulative effect of oral diseases [27]. The main limitation of the present study was its cross-sectional design. The missing defensin data of younger age groups were also a limitation; however, a comparison of our current results with those of working-aged (40–60 years) adults [21] was feasible. The methods for the saliva collection and defensin measurements were similar in both studies, which allowed the advantage of reliable comparison. Finally, considering that HNP-1 is the major HNP present in the oral cavity, the salivary HNP-2 and HNP-3 levels were not studied here. Indeed, it has been suggested that HNP-2 is produced by the post-translational proteolytic cleavage of HNP-3 [28]. Future longitudinal studies are necessary to understand the shifts in salivary defensin levels in relation to age, systemic conditions, lifestyle, and oral inflammatory status.

## 5. Conclusions

In the elderly, salivary defensins HNP-1 and hBDs differ in terms of their correlation to the extent of periodontal destruction, as defined by the number of pocket teeth. Further studies are needed to explain the differences in the regulation of these two groups of oral antimicrobial defensins with aging. In future, it would be beneficial to measure the activators and suppressors of both α- and β-defensins to explain the underlying mechanisms of antimicrobial peptide stimulation in the elderly. The elevated HNP-1 levels observed in the elderly having advanced periodontal destruction may indicate its diagnostic predictive value for further progression of disease.

## Figures and Tables

**Table 1:** The characteristics of the study population. Age and number of teeth are presented as mean ± standard deviation. Males, level of education, and smokers are given as percentages. Body mass indexes (BMI, kg/m^2^) are presented as medians and interquartile ranges in parenthesis.

		Control *n* = 17	PerioA *n* = 55	PerioB *n* = 33	PerioC *n* = 29	Edentulous *n* = 41	*p*
Age, years		74.7 ± 5.1	73.8 ± 7.1	73.8 ± 6.4	71.8 ± 4.6	77.2 ± 6.6	0.009
Male %		17.6%	32.7%	45.5%	65.5%	36.6%	0.010
Level of education %	Basic	41.2%	49.1%	30.3%	34.5%	78.0%	0.011
	Upper secondary	17.6%	23.6%	36.4%	20.7%	14.6%	
	Higher	41.2%	27.3%	33.3%	44.8%	7.3%	
Smokers %		0%	10.9%	15.2%	20.7%	12.5%	0.511
Number of teeth		13.3 ± 8.7	15.6 ± 8.8	21.5 ± 6.7	23.5 ± 3.5	0 ± 0	<0.001
Body mass index		27.0 (7.19)	26.9 (6.0)	27.2 (4.7)	27.1 (4.4)	26.1 (5.7)	0.942

Control: pocket-free individuals, PerioA: individuals having 1–6 pocket teeth, PerioB: individuals having 7–13 pocket teeth, PerioC: individuals having at least 14 pocket teeth.

**Table 2:** Unadjusted and adjusted associations between salivary HNP-1 levels and an increased number of teeth with PPD ≥ 4 mm.

	Unadjusted	Adjusted
Salivary HNP-1	β = 0.424, *p* < 0.001	β = 0.282, *p* = 0.003

The model is adjusted for age, gender, level of education, and number of teeth.

## Data Availability

The data of this study are available from the corresponding author upon reasonable request.
